# Relationship between serum lipid levels and ischemic stroke in patients with atrial fibrillation: a nested case–control study based on the China Atrial Fibrillation Registry

**DOI:** 10.1186/s12872-021-02237-6

**Published:** 2021-09-08

**Authors:** Fei Li, Xin Du, Liu He, Chao Jiang, Shijun Xia, Changsheng Ma, Jianzeng Dong

**Affiliations:** Department of Cardiology, Beijing Anzhen Hospital, National Clinical Research Center for Cardiovascular Diseases, Capital Medical University, 2 Anzhen Road, Chaoyang District, Beijing, China

**Keywords:** Stroke, Risk, Hyperlipidemia, Triglycerides, Atrial fibrillation

## Abstract

**Background:**

Atrial fibrillation (AF) is an important risk factor for acute ischemic stroke.

**Methods:**

A nested case–control study was conducted among patients diagnosed with AF, whose information was acquired from the prospective China Atrial Fibrillation Registry (China-AF), from August 2011 to December 2018.

**Results:**

This study compared patients with stroke group (n = 145) with a matched control group (n = 577). Demographic data were similar except for body mass index (BMI), diastolic blood pressure (DBP) which were higher, and new oral anticoagulant (NOAC) treatment rate which was lower in the stroke group (all *P* < 0.05). Baseline median [IQR] levels of including triglyceride (TG) were higher in the stroke group (21.96 [16.74, 21.52], mg/dL) than the control group (19.62 [14.76, 27.36], mg/dL) (*P* = 0.012), while the total cholesterol (TC), low-density lipoprotein cholesterol (LDL-C) and high-density lipoprotein cholesterol (HDL-C) were similar between the two groups. Elevated TG and HDL-C were positively associated with ischemic stroke (OR 1.01, 95% CI 1.00–1.02, *P* = 0.032; OR 1.03, 95% CI 1.00–1.05, *P* = 0.025), after adjustment for BMI, systolic blood pressure, DBP, CHA_2_DS_2_-VASc score, HAS-BLED score, NOAC, LDL-C and HDL-C. However, NOAC (OR 0.20, 95% CI 0.05–0.84, *P* = 0.029) could decrease the likelihood of ischemic stroke in patients with AF. In subgroup analysis, higher TG level remained significantly associated with ischemic stroke for AF patients without a history of smoking (OR 1.26, 95% CI 1.02–1.55, *P* = 0.028).

**Conclusion:**

Higher level of TG and HDL-C were positively associated with ischemic stroke in patients with AF.

**Supplementary Information:**

The online version contains supplementary material available at 10.1186/s12872-021-02237-6.

## Background

Around 16.9 million people suffer a stroke each year and the global burden of stroke is increasing dramatically [1]. China has the biggest stroke burden in the world and about 70% of cases are due to ischemic stroke, when a blood vessel supplying the brain becomes obstructed [2]. Therefore, ischemic stroke is a major cause of morbidity and mortality for Chinese adults. Atrial fibrillation (AF) is an important risk factor for an acute ischemic stroke [3]. Strokes related to AF are also associated with higher mortality and more severe neurologic disability [4]. So, patients with AF are encouraged to consider prevention measures such as oral anticoagulation therapy and modifying their diet and behavior [5].

Dyslipidemia is another well-established risk factor for stroke [6]. Dyslipidemia is implicated in the development of atherosclerosis and coronary heart disease, which are both closely related to AF development [7]. However, while high levels of low-density lipoprotein cholesterol (LDL-C) and low levels of high-density lipoprotein cholesterol (HDL-C) are closely associated with developing coronary artery disease, [7] the relationship with AF is less obvious. Meta-analysis suggests an inverse relationship between serum total cholesterol (TC), LDL-C, and HDL-C levels and AF risk, although there was no significant association between triglyceride (TG) levels and incident AF [8]. However, because both AF and dyslipidemia are independent risk factors for stroke there is a theoretical possibility that patients who experience both may be at greater risk of ischemic stroke.

One study, identified a high level of LDL-C as an independent risk factor for stroke in patients with AF [9]. Another study found that statin use by patients with AF prior to stroke reduced oxidized low-density lipoprotein levels and improved outcomes after stroke [10]. However, large-scale studies are lacking, so it is not clear whether blood lipid levels should be lowered in all patients with AF and if so, the appropriate level of blood lipids needs to be established with quantitative evidence.

Ischemic stroke is a major adverse event in patients with AF, which affects the prognosis of patients [11]. If AF is combined with dyslipidemia, there is a theoretical superposition effect, which further aggravates the occurrence of ischemic stroke [12]. In addition to directly causing ischemic stroke, blood lipid levels also reflect the level of inflammation in patients [13, 14], and studies have shown that blood lipid levels are related to the occurrence of atrial fibrillation [15]. Therefore, in addition to the superposition effect of AF with dyslipidemia, there may also be a synergistic effect, by which this combination further increases the incidence of ischemic stroke. Blood lipid is a risk factor for stroke, but medical personnel primarily focus on controlling the ventricular rate and anticoagulation in patients with AF. Elevated blood lipid levels can cause endothelial inflammation, theoretically increasing the likelihood of forming thrombosis. Thus far, it is unknown whether or not patients with AF need blood lipid management.

Therefore, our study aimed to explore the correlation between baseline blood lipid level and the occurrence of ischemic stroke in patients with AF. The China Atrial Fibrillation Registry (China-AF) is a prospective, multicenter, hospital-based registry of participants diagnosed with AF. This registry, therefore, provides a large amount of real data that can be analyzed and explored, to provide reference levels and evidence for the control of blood lipids in patients with AF.

## Methods

### Patients

Patients diagnosed with AF from August 2011 to December 2018 were investigated from data from the China-AF study. Details of the China-AF study design have been published previously [16, 17]. Briefly, the China-AF study is a prospective, multicenter, hospital-based registry of patients with AF from 31 hospitals providing AF treatment in Beijing. Beijing Anzhen Hospital, China provided study coordination and site management.

The inclusion criteria were as follows: (1) age over 18 years old; (2) AF was accurately recorded on electrocardiogram (ECG) or Holter within 6 months; (3) no lipid-lowering drugs had been taken before. The exclusion criteria were as follows: (1) follow-up less than 6 months; (2) patients after radiofrequency ablation; (3) lack of blood lipid records; (4) history of cerebral infarction.

This study complied with the standards required for an observational study. The procedures followed were in accordance with the Declaration of Helsinki (2008). Ethical approval was obtained from the Human Research Ethics Committees at Beijing Anzhen Hospital. Reference number: 20110610. The ethics review boards in Beijing Anzhen Hospital, Capital Medical University approved their participation. Each patient provided written informed consent for long-term follow-up. This study was registered (ChiCTR-OCH-13003729.).

### Study design

This was a nested case–control study. Patients who were eligible for inclusion in the study and experienced ischemic stroke during the follow-up period were allocated to the stroke group. The remaining population was then matched according to age (difference of < 2 years), gender, and the same follow-up time (in months) with a 1:4 ratio of patients in the stroke group to patients in the non-stroke group.

### Clinical data collection and examination method

The baseline data of the patients included age, gender, body mass index (BMI), education background, history of smoking, history of drinking, medical insurance, past history, and medication records, as well as blood pressure, heart rate, and biochemical indicators of the patients recorded at the time of visiting the hospital. Among the biochemical indexes, blood lipid related indexes included TG, TC, HDL-C, and LDL-C. The blood tests were taken at the baseline of the China-AF registry with the requirement of fasting. The information about blood lipids was collected before the onset of stroke without traditional lipid-lowering medication, which could avoid reverse causality inference.

At the same hospital visit, the patients completed a transthoracic echocardiography (TTE) examination, and left atrial inner diameter (LA), left ventricular end-diastolic dimension (LVEDD), left ventricular function (LVEF) were recorded. The type of AF was distinguished between paroxysmal AF and non-paroxysmal AF. The patients’ CHA_2_DS_2_-VASc and HAS-BLED scores [18] were evaluated.

### Definitions and follow-up

Patients were followed up at 3 months, 6 months, and every 6 months thereafter by trained staff at the outpatient clinics or through telephone interview.

### Statistical analysis

During the follow-up period each case that experienced an ischemic stroke was matched with four controls with similar follow-up time. The matching ensured that they had the same gender and were within 2 years of age. The continuous data in normal distribution were presented as mean ± standard deviation, and skewed data were presented as median (interquartile range, IQR). The categorical data were presented as frequency (percentage). Student’s *t* test or Mann–Whitney *U* test, and chi-square test was used in comparison between two groups.

Conditional logistic regression models were conducted, taking the occurrence of an ischemic stroke event as the dependent variable, and taking each serum lipid level as an independent variable, adjusted for confounders with important clinical concern. Subgroup analysis was also performed using conditional logistic regression models to explore the associations of serum lipid level and ischemic stroke by different BMI groups, smoking status, alcohol assumption status, different type of AF, history of cardiovascular disease and metabolic disease, different levels of CHA_2_DS_2_-VASc scores and HAS-BLED scores; heterogeneity across subgroups was tested by introducing an interaction term of serum lipid level and the grouping variable into models. Two-sided *P* < 0.05 was considered statistically significant. All statistical analysis was performed using SAS version 9.4 (SAS Institute Inc., Cary, NC, USA).

## Results

### Baseline characteristics of the study population

**Fig. 1:**
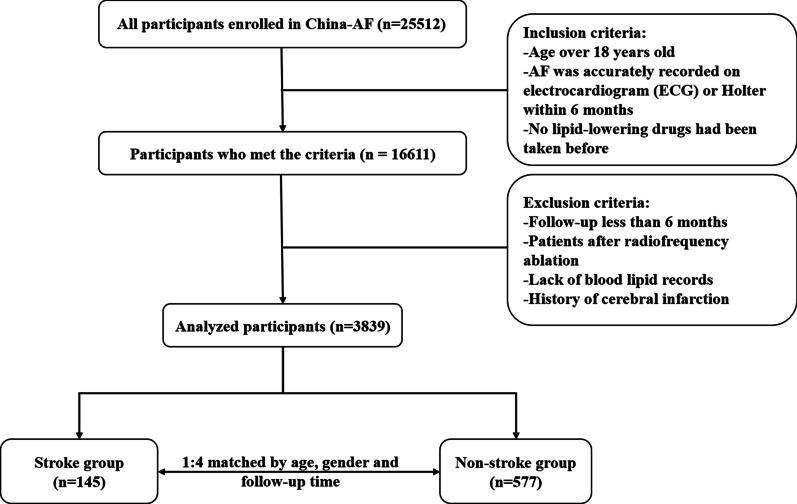
Patient selection flowchart

Between August 2011 to December 2018, 16,611 patients were enrolled in the study according to the inclusion criteria. Of these, 2234 patients were excluded due to having less than 6 months of follow-up; 6326, due to previously having undergone radiofrequency ablation; 3675, due to the lack of blood lipid data; and 537, due to the history of cerebral infarction. Finally, 3839 patients were considered for inclusion in this study. Of this population, 145 were eligible for allocation in the stroke group, and the remaining 577 were identified eligible for allocation to the non-stroke control group after matching. Figure 1 presents the patient selection flowchart of this study.

Table 1 presents the baseline data of the study population. Apart from the differences in the BMI, diastolic blood pressure (DBP), and new oral anticoagulant (NOAC) utilization rate between the two groups, the other demographic baseline data were similar between the two groups.

**Table 1:** Baseline data of patients

Characteristics	Stroke (n = 145)	Non-stroke (n = 577)	*P* value
*Demographics*			
Age (years), mean ± SD	70.5 ± 10.7	70.5 ± 10.7	0.983
Gender (male), n (%)	78 (53.8)	309 (53.5)	0.959
BMI, mean ± SD	25.5 ± 3.8	24.8 ± 3.4	0.033
Normal (< 24 kg/m^2^), n (%)	40 (31.0)	211 (40.8)	0.014
Overweight (24–28 kg/m^2^), n (%)	56 (43.4)	226 (43.7)	–
Obese (BMI ≥ 28 kg/m^2^), n (%)	33 (25.6)	80 (15.5)	–
Current Smoking, n (%)	25 (17.2)	77 (13.3)	0.229
Current Drinking, n (%)	25 (17.2)	99 (17.2)	0.981
High school completion, n (%)	39 (26.9)	134 (23.2)	0.354
Partial or full health insurance, n (%)	134 (92.4)	529 (91.7)	0.773
*Medical history, n (%)*			
Cardiovascular System	50 (34.5)	191 (33.1)	0.768
Cerebrovascular system	6 (4.1)	18 (3.1)	0.603
Respiratory system	1 (0.7)	9 (1.6)	0.696
Metabolic disease	50 (34.5)	185 (32.1)	0.620
*Vital signs*			
SBP (mmHg), mean ± SD	132.0 ± 17.2	129.0 ± 17.0	0.058
DBP (mmHg), mean ± SD	79.0 ± 12.9	76.5 ± 11.0	0.029
Heart rate (bpm), median (IQR)	80 (65,94)	78 (68,92)	0.969
*Laboratory tests*			
FBG (mmol/L), median (IQR)	5.5 (4.9,6.7)	5.4 (4.9,6.2)	0.252
eGFR (mL/min/1.73 m^2^), median (IQR)	96.0 (80.8,113.9)	100.8 (81.3,121.22)	0.284
BNP (pg/mL), median (IQR)	401.5 (139.0,860.3)	221.0 (94.0,709.3)	0.265
NT-pro-BNP (pg/mL), median (IQR)	1423.0 (682.0,3885.0)	1129.0 (521.2,2295.0)	0.233
*TTE*			
LA (mm), median (IQR)	41.0 (37.0,46.3)	42.0 (37.0,46.0)	0.996
LVEDD (mm), median (IQR)	47.0 (45.0,52.0)	48.0 (44.5,52.0)	0.545
LVEF (%), median (IQR)	62.0 (55.0,67.0)	63.0 (58.0,68.0)	0.069
*Atrial fibrillation duration (year), median (IQR)*	2.5 (0.6,5.9)	2.9 (0.8,8.3)	0.168
CAD	18 (12.4)	47 (8.2)	0.108
CKD	11 (7.6)	35 (6.1)	0.503
*Atrial fibrillation type, n (%)*			0.364
Paroxysmal	62 (42.8)	271 (47.0)	
Non-paroxysmal	83 (57.2)	306 (53.0)	
*Atrial fibrillation type, n (%)*			0.212
Valvular	1 (0.7)	15 (2.6)	
Non-valvular	144 (99.3)	562 (97.4)	
*CHA*_*2*_*DS*_*2*_*-VASc score, n (%)*	3.0 (2.0–4.0)	3.0 (2.0–4.0)	0.628
Low/medium risk (≤ 1)	24 (16.6)	114 (19.8)	0.380
High risk (≥ 2)	121 (83.4)	463 (80.2)	
HAS-BLED score, median (IQR)n (%)	2.0 (2.0–3.0)	2.0 (2.0–3.0)	0.438628
Low risk (< 3)	84 (57.9)	347 (60.1)	0.628
High risk (≥ 3)	61 (42.1)	230 (39.9)	
*Contaminant medications, n (%)*			
Antiarrhythmic drugs	16 (11.0)	94 (16.3)	0.123
Ventricular rate control	91 (62.8)	341 (59.1)	0.422
*Antithrombotic drugs*			
Aspirin	66 (45.5)	241 (41.8)	0.453
Warfarin	47 (32.4)	186 (32.2)	0.967
NOAC	2 (1.4)	39 (6.8)	0.012

BMI, body mass index; SBP, systolic blood pressure; DBP, diastolic blood pressure; FBG, fasting blood glucose; eGFR, estimated glomerular filtration rate; BNP, B-type natriuretic peptide; NT-pro-BNP, N terminal pro-BNP; TTE, transthoracic echocardiography; LA, left atrial inner diameter; LVEDD, left ventricular end-diastolic dimension; LVEF, left ventricular ejection fraction; CAD, coronary artery disease; CKD, chronic kidney disease; NOAC, new oral anticoagulants

### Comparison of blood lipid levels

Table 2 presents the difference in the blood lipid levels between the two groups. The TG level of patients was significantly higher in the stroke group (21.96 [16.74, 21.52]) than in the non-stroke group (19.62 [14.76, 27.36]; *P* = 0.012). There was no significant difference in the TC, LDL-C, and HDL-C between the groups.

**Table 2:** Comparison of blood lipid levels between the stroke and non-stroke groups

Lipid-related parameters	Stroke (n = 145)	Non-stroke (n = 577)	*P* value
TG (mg/dLmmol/L), median (IQR)	21.961.22 (16.740.93, 21.521.64)	19.621.09 (14.760.82, 27.361.52)	0.012
TC (mg/dLmmol/L), median (IQR)	79.924.44 (70.743.93, 94.505.25)	79.024.39 (66.783.71, 89.104.95)	0.137
LDL-C (mg/dLmmol/L), median (IQR)	49.322.74 (36.182.01, 56.343.13)	46.982.61 (37.082.06, 54.363.02)	0.3243
HDL-C (mg/dLmmol/L), median (IQR)	21.061.17 (17.640.98, 25.021.39)	21.241.18 (17.280.96, 25.021.39)	0.701

TG, triglyceride; TC, total cholesterol; LDL-C, low-density lipoprotein cholesterol; HDL-C, high-density lipoprotein cholesterol

### Multivariable analysis of factors related to ischemic stroke

Multivariable analysis of the factors associated with the occurrence of ischemic stroke in patients with AF is shown in Table 3 for TG, and Additional file 1: Tables S1, S2 and S3 for TC/LDL-C/HDL-C. The results suggested that the elevation of TG and HDL-C increased the possibility of the occurrence of ischemic stroke in patients with AF (OR 1.05, 95% CI 1.00–1.02, *P* = 0.032; OR 1.03, 95% CI 1.00–1.05, *P* = 0.025), indicating that it was an independent risk factor. However, NOAC (OR 0.20, 95% CI 0.05–0.84, *P* = 0.029) decreased the likelihood of ischemic stroke in patients with AF. The TC and LDL-C showed no independent association with the occurrence of ischemic stroke in AF patients.

**Table 3:** Conditional logistic regression analysis of TG level and ischemic stroke

	Univariable analysis			Multivariable analysis		
	OR	95% CI	*P* value	OR	95% CI	*P* value
TG (mg/dLmmol/L)	1.0122	1.003–1.0244	0.024	1.0120	1.001–1.0242	0.0325
BMI	1.06	1.01–1.12	0.032	1.05	0.99–1.11	0.089132
SBP	1.01	0.99–1.02	0.066	1.00	0.99–1.02	0.56216
DBP	1.02	1.00–1.04	0.020	1.01	0.99–1.03	0.14780
*CHA*_*2*_*DS*_*2*_*-VASc score*						
Low/medium risk (≤ 1)	Reference	–	–	Reference	–	–
High risk (≥ 2)	1.66	0.78–3.51	0.187	1.327	0.602–2.938	0.49036
*HAS-BLED score*						
Low risk (< 3)	Reference	–	–	Reference	–	–
High risk (≥ 3)	1.11	0.73–1.69	0.637	0.991.01	0.635–1.56	0.9750
NOAC	0.19	0.04–0.79	0.022	0.20	0.05–0.84	0.029
LDL-C (mg/dL)	1.01	0.99–1.02	0.318	1.01	0.99–1.02	0.426
HDL-C (mg/dL)	1.02	0.99–1.04	0.163	1.03	1.00–1.05	0.025

BMI, body mass index; SBP, systolic blood pressure; DBP, diastolic blood pressure; TG, triglyceride; TC, total cholesterol; NOAC, new oral anticoagulant; LDL-C, low-density lipoprotein cholesterol; HDL-C, high-density lipoprotein cholesterol

### Subgroup analysis

The results of the subgroup analysis (Table 4) showed, after adjusting for confounders, that TG was associated with the occurrence of ischemic stroke in AF patients without a history of smoking (OR 1.01, 95% CI 15.12–28.08, *P* = 0.034), patients ≥ 70 years of age (OR 1.02, 95% CI 1.00–1.03; *P* = 0.036); and AF patients with CHA_2_DS_2_-VASc score ≥ 2 (OR 1.01, 95% CI 1.00–1.03, *P* = 0.047). However, no heterogeneity across subgroups were detected, so it cannot be concluded that the association between blood lipids and stroke in different subgroups was different.

**Table 4:** Subgroup analysis of the associations of TG level and ischemic stroke

Subgroups	TG (mgmol/dL), median (IQR)	OR	95% CI	*P* value	*P* for interaction
*BMI*					0.223263
Normal (< 24 kg/m^2^)	18.181.01 (14.220.79, 1.3624.48)	1.0128	0.9769–1.052.38	0.556434^a^	
Overweight (24–28 kg/m^2^)	20.521.14 (15.300.85, 1.6229.16)	1.2101	0.994–1.0357	0.13443^a^	
Obese (BMI ≥ 28 kg/m^2^)	24.121.34 (18.181.01, 1.7932.22)	0.981.07	0.9130–3.821.06	0.919^a^629^a^	
*Age*					0.306
< 70	22.68 (16.20, 31.50)	1.01	0.99–1.02	0.547^a^	
≧70	18.90 (14.49, 25.65)	1.02	1.00–1,03	0.036^a^	
*Smoking*					0.21670
Yes	18.361.02 (0.14.9483, 28.801.60)	0.991.02	0.9341–1.062.53	0.822965^a^	
No	20.521.14 (15.120.84, 1.5628.08)	1.0126	1.003–1.0355	0.03428^a^	
*Drinking*					0.73148
Yes	19.891.11 (14.850.83,1.6128.89)	1.0239	0.9763–1.083.07	0.366421^a^	
No	20.251.13 (15.120.84, 1.5627.90)	1.2101	0.991.00–1.0247	0.04961^a^	
*Atrial fibrillation type*					0.76370
Paroxysmal	21.421.19 (15.840.88,1.6229.16)	1.0232	0.995–1.0482	0.130094^a^	
Non-paroxysmal	18.901.05 (14.760.82, 1.4726.46)	1.0119	0.992–1.0354	0.216181^a^	
*Cardiovascular disease*					0.071062
Yes	18.001.00 (13.860.77,1.3724.66)	1.0255	0.9988–1.062.75	0.265132^a^	
No	21.421.19 (16.020.89, 1.6229.16)	1.0111	0.9987–1.4202	0.339403^a^	
*Metabolic disease*					0.462380
Yes	21.601.20 (16.020.89, 1.7631.68)	1.0376	1.007–1.062.91	0.106027^a^	
No	19.441.08 (0.8214.76, 1.4926.82)	1.018	0.9975–1.0354	0.62990^a^	
*CHA*_*2*_*DS*_*2*_*-VASc score*					0.29655
Low/medium risk (≤ 1)	21.601.20 (15.30.85, 1.6830.24)	1.014	0.9644–1.062.47	0.804922^b^	
High risk (≥ 2)	1.0919.62 (15.120.84, 1.5227.27)	1.0125	1.002–1.0352	0.04731^b^	
*HAS-BLED score*					0.477544
Low risk (< 3)	21.421.19 (15.300.85, 1.6128.98)	1.0112	0.990–1.0241	0.265311^c^	
High risk (≥ 3)	18.901.05 (14.580.81, 1.4726.46)	1.3101	0.9881–1.042.13	0.503279^c^	

BMI, body mass index; SBP, systolic blood pressure; DBP, diastolic blood pressure; TG, triglyceride

^a^Adjusted by BMI, SBP, DBP, CHA_2_DS_2_-VASc score, and HAS-BLED score, NOAC, LDL and HDL

^b^Adjusted by BMI, SBP, DBP, and HAS-BLED score, NOAC, LDL and HDL

^c^Adjusted by BMI, SBP, DBP, and CHA_2_DS_2_-VASc score, NOAC, LDL and HDL

## Discussion

In order to discover whether blood lipid levels are associated with ischemic stroke in patients with AF, this study investigated baseline blood lipid levels and other clinical factors in patients with AF who experienced ischemic stroke and compared them with the values in matched patients with AF who did not experience a stroke. The results show that TG levels were higher in the stroke group than the non-stroke group, but TC, LDL-C and HDL-C were similar. Elevated TG and HDL-C was also associated with ischemic stroke. Subgroup analysis suggested TG remained associated with ischemic stroke in patients with AF over 70 years old, in patients with AF without a history of smoking, and in patients with AF with CHA_2_DS_2_-VASc score ≥ 2. Therefore, patients with AF and high TG should consider methods to lower their TG levels, especially if they are over 70 years old, have not smoked, or have CHA_2_DS_2_-VASc score ≥ 2.

The relationship between blood lipid levels and the risk of ischemic stroke in patients with AF may be complex. It is generally established that the risk of atherosclerosis increases with higher LDL-C [19]. In general populations at risk of ischemic stroke, statin therapy trials have shown that lowering LDL-C by 1 mmol/L reduces the risk of both ischemic stroke and coronary heart disease by about 20% [20]. On the other hand, observational studies have found stronger effects of LDL-C on coronary heart disease than ischemic stroke [21]. These differences highlight the heterogeneity in the effects of cholesterol on different subtypes of ischemic stroke [22]. In Chinese populations, the LDL-C level tends to be lower than in Western populations. Nevertheless, lowering LDL-C in Chinese populations also showed a benefit in preventing ischemic stroke, even below levels considered a risk in Western populations [23]. In China, a previous study suggested a high level of LDL-C was an independent risk factor for stroke in patients with AF [9]. While another study found that reducing oxidized low-density lipoprotein levels with statins prior to stroke reduced and improved outcomes after stroke in patients with AF [10]. However, this study did not find an independent relationship between high LDL-C level and ischemic stroke risk. This result may be influenced by the results of a study that showed patients with AF have reduced LDL-C levels compared to healthy controls [7].

Low HDL-C was reportedly associated with increased possibility the ischemic stroke [24, 25]. However, most recent meta-analysis of the modifiable and non-modifiable risk factors in the Asian population showed that HDL-C was not significantly associated with ischemic stroke [26], while in the review by Kloska et al. decreased HDL-C have been identified as risk factor and predictor of cardiovascular disease, including stroke, in Caucasian population [27]. Conversely, study by Yang found that higher level of HDL was independent indicators of newly-detected AF [28], while Lee et al., also in the Asian population, reported that high TG and low HDL levels were associated with CHD risk (including risk of ischemic stroke) only in participants with an LDL-C level of ≥ 130 mg/dL, but this was not observed in those participants with lower LDL-C levels [29]. The results of our study suggest that the elevation of HDL-C might increase the possibility of the ischemic stroke in patients with AF. This result needs further confirmation, as it may be influenced by non-categorized HDL-C and a lower portion of NOAC users in the stroke group. This difference might lead to some bias. On the one hand, this may be because NOAC is expensive and patients with stroke already incur healthcare costs. On the other hand, perhaps fewer patients use NOAC due to non-compliance, resulting in an uneven distribution. Since the different distribution of NOAC users could introduce bias, NOAC was included as a covariate in the conditional logistic regression models. Still, regarding NOAC therapy, our findings are in line with previous study, reporting decreased ischemic stroke, remarkably low rate of systemic embolism and bleeding in AF patients [30], which suggests that patients receiving NOAC might have lower rate of ischemic stroke. The results of this study did suggest that TG levels were an important factor in ischemic stroke in patients with AF. High TG levels increase the chance of cardiovascular events due to increased atherosclerosis [31]. TG may also stimulate atherogenesis through availability of excessive free fatty acids, production of proinflammatory cytokines, fibrinogen, coagulation factors and impairment of fibrinolysis [32]. Some studies suggest a direct relationship between TG and stroke [33]. Elevated TG caused in relation to genetic factors may not affect stroke risk [34]. Paradoxically, even low TG levels have been shown to play a role in ischemic stroke. Jain et al. studied patients with acute ischemic stroke and found that low TG level was associated with a worse prognosis [35]. Ryu et al. reported that low serum TG is an independent predictor of mortality after ischemic stroke by non-cardioembolic causes [36]. To date, however, the relationship between TG and ischemic stroke in patients with AF has not been fully established. Thus, the results of this study suggest the need to further investigate the relationship between TG and ischemic stroke in patients with AF. TG-lowering methods including diet and lifestyle changes might be considered. When subgroup analysis was applied, the effect of high TG on ischemic stroke risk was maintained in patients over 70 years old but not in the younger group, those without a history of smoking, and those with CHA_2_DS_2_-VASc score ≥ 2. Therefore, patients in these particular subgroups may find that efforts to reduce TG levels yield the most benefit in terms of reduced incidence of stroke.

This study has some limitations. As this study was performed in a Chinese population, its results cannot entirely be generalized outside of this context. Further studies should be performed with patients of a different ethnicity. Of the total of 25,512 patients with AF enrolled in the China-AF study, only 3839 patients were analyzed and only 722 patients were analyzed in both the groups. Therefore, the potential for selection bias must be considered as a limitation. Although the groups were matched, there were some differences between them in terms of the BMI, DBP, and NOAC use, which may have introduced some bias into the results. While TG was identified as being associated with ischemic stroke, we did not identify a clinically meaningful cutoff value and the effect of decreasing TG levels was not investigated. Further prospective, large-scale randomized clinical trials are needed to further provide high-level evidence.

## Conclusion

This study showed that patients with AF who experienced ischemic stroke had higher TG levels compared to matched patients with AF without stroke; however, the TC, LDL-C, and HDL-C levels were similar between both patient groups. Multivariate analysis showed that TG, HDL-C and NOAC were associated with ischemic stroke in patients with AF. TG was also found in subgroup analysis of patients with AF for those aged over 70 years, without a history of smoking, and with CHA_2_DS_2_-VASc score ≥ 2. These results suggest that studies to investigate the benefit to patients with AF in decreasing TG should be undertaken.

## Supplementary Information

**Additional file 1. Supplementary Table 1**. Multivariable conditional logistic regression analysis for TC related to ischemic stroke. **Supplementary Table 2**. Multivariable conditional logistic regression analysis for LDL-C related to ischemic stroke. **Supplementary Table 3**. Multivariable conditional logistic regression analysis for HDL-C related to ischemic stroke.

## Data Availability

The datasets used and/or analyzed during the current study are available from the corresponding author on reasonable request.

## Abbreviations

AF: Atrial fibrillation
China-AF: China Atrial Fibrillation Registry
BMI: Body mass index
DBP: Diastolic blood pressure
LDL-C: Low-density lipoprotein cholesterol
HDL-C: High-density lipoprotein cholesterol
TC: Total cholesterol
TG: Triglyceride
ECG: Electrocardiogram
TTE: Transthoracic echocardiography
LA: Left atrial inner diameter
LVEDD: Left ventricular end-diastolic dimension
LVEF: Left ventricular function
