# Supplementary material for: Relationship between serum lipid levels and ischemic stroke in patients with atrial fibrillation: a nested case–control study based on the China Atrial Fibrillation Registry
Source: BMC Cardiovasc Disord. 2021 Sep 8;21:424. doi: 10.1186/s12872-021-02237-6 (PMC8425053; doi:10.1186/s12872-021-02237-6)
Supplement: Supplementary file 1 — Additional file 1. Supplementary Table 1. Multivariable conditional logistic regression analysis for TC related to ischemic stroke. Supplementary Table 2. Multivariable conditional logistic regression analysis for LDL-C related to ischemic stroke. Supplementary Table 3. Multivariable conditional logistic regression analysis for HDL-C related to ischemic stroke. [file 12872_2021_2237_MOESM1_ESM.docx]

**Relationship between serum lipid levels and ischemic stroke in patients with atrial fibrillation: a nested case-control study based on the China Atrial Fibrillation Registry**

**Running title:** AF blood lipid levels and ischemic stroke risk

Fei Li, Xin Du, Liu He, Chao Jiang, Shijun Xia , Changsheng Ma, Jianzeng Dong^*^

Department of Cardiology, Beijing Anzhen Hospital, Capital Medical University; National Clinical Research Center for Cardiovascular Diseases, Beijing, China

**^*^Corresponding Author:**

Jianzeng Dong

Department of Cardiology, Beijing Anzhen Hospital, Capital Medical University; National Clinical Research Center for Cardiovascular Diseases, 2 Anzhen Road, Chaoyang District, Beijing, China

Tel: 010-64412431

E-mail: jzdong@ccmu.edu.cn

**Supplementary Table 1.** Multivariable conditional logistic regression analysis for TC related to ischemic stroke

| Characteristics | Multivariable analysis | | |
| --- | --- | --- | --- |
|  | OR | 95% CI | *P* value |
| TC (mg/dL) | 1.00 | 0.99-1.02 | 0.199 |
| BMI | 1.05 | 0.99-1.11 | 0.124 |
| SBP | 1.00 | 0.99-1.02 | 0.551 |
| DBP | 1.01 | 0.99-1.03 | 0.161 |
| CHA_2_DS_2_-VASc score |  |  |  |
| Low/medium risk (≤1) | Reference | / | / |
| High risk (≥2) | 1.34 | 0..61-2.94 | 0.470 |
| HAS-BLED score |  |  |  |
| Low risk (<3) | Reference | / | / |
| High risk (≥3) | 0.96 | 0.62-1.50 | 0.860 |
| NOAC | 0.20 | 0.05-0.86 | 0.031 |
| TG | 1.00 | 1.00-1.02 | 0.122 |

BMI: body mass index; SBP: systolic blood pressure; DBP: diastolic blood pressure; TC: total cholesterol; NOAC: new oral anticoagulant; TG: triglyceride.

**Supplementary Table 2.** Multivariable conditional logistic regression analysis for LDL-C related to ischemic stroke

| Characteristics | Multivariable analysis | | |
| --- | --- | --- | --- |
|  | OR | 95% CI | *P* value |
| LDL-C (mg/dL) | 1.01 | 0.99-1.02 | 0.426 |
| BMI | 1.05 | 0.99-1.11 | 0.089 |
| SBP | 1.00 | 0.99-1.02 | 0.562 |
| DBP | 1.01 | 0.99-1.03 | 0.147 |
| CHA_2_DS_2_-VASc score |  |  |  |
| Low/medium risk (≤1) | Reference | / | / |
| High risk (≥2) | 1.32 | 0.60-2.93 | 0.490 |
| HAS-BLED score |  |  |  |
| Low risk (<3) | Reference | / | / |
| High risk (≥3) | 0.99 | 0.63-1.56 | 0.975 |
| NOAC | 0.20 | 0.05-0.84 | 0.029 |
| TG (mg/dL) | 1.01 | 1.00-1.02 | 0.032 |
| HDL-C (mg/dL) | 1.03 | 1.00-1.05 | 0.025 |

BMI: body mass index; SBP: systolic blood pressure; DBP: diastolic blood pressure; LDL-C: low-density lipoprotein cholesterol; NOAC: new oral anticoagulant; TG: triglyceride; HDL-C: high-density lipoprotein cholesterol.

**Supplementary Table 3.** Multivariable conditional logistic regression analysis for HDL-C related to ischemic stroke

| Characteristics | Multivariable analysis | | |
| --- | --- | --- | --- |
|  | OR | 95% CI | *P* value |
| HDL-C (mg/dL) | 1.03 | 1.00-1.05 | 0.025 |
| BMI | 1.05 | 0.99-1.12 | 0.089 |
| SBP | 1.00 | 0.99-1.02 | 0.562 |
| DBP | 1.01 | 0.99-1.03 | 0.147 |
| CHA_2_DS_2_-VASc score |  |  |  |
| Low/medium risk (≤1) | Reference | / | / |
| High risk (≥2) | 1.32 | 0.60-2.93 | 0.490 |
| HAS-BLED score |  |  |  |
| Low risk (<3) | Reference | / | / |
| High risk (≥3) | 0.99 | 0.63-1.56 | 0.975 |
| NOAC | 0.20 | 0.05-0.84 | 0.029 |
| TG (mg/dL) | 1.01 | 1.00-1.02 | 0.032 |
| LDL-C (mg/dL) | 1.01 | 0.99-1.02 | 0.426 |

BMI: body mass index; SBP: systolic blood pressure; DBP: diastolic blood pressure; HDL-C: high-density lipoprotein cholesterol; NOAC: new oral anticoagulant; TG: triglyceride; LDL-C: low-density lipoprotein cholesterol.
